# Cohort analysis of the state of female genital cutting in Nigeria: prevalence, daughter circumcision and attitude towards its discontinuation

**DOI:** 10.1186/s12905-021-01324-2

**Published:** 2021-04-29

**Authors:** Babatunde M. Gbadebo, Adetokunbo T. Salawu, Rotimi F. Afolabi, Mobolaji M. Salawu, Adeniyi F. Fagbamigbe, Ayo S. Adebowale

**Affiliations:** Department of Epidemiology and Medical Statistics, Faculty of Public Health, College of Medicine, University of Ibadan, Ibadan, Nigeria

**Keywords:** Cohort analysis, Complications, Female genital cutting, Harmful traditional practices, Nigeria

## Abstract

**Background:**

Female genital cutting (FGC) inflicts life-long injuries on women and their female children. It constitutes a violation of women’s fundamental human rights and threats to bodily integrity. Though decreasing, the practice is high and widespread in Nigeria despite efforts towards its eradication. This study was conducted to perform cohort analysis of the state of FGC between the years 2009 and 2018 in Nigeria.

**Results:**

The study found that that FGC has reduced over the years from 56.3% among the 1959–1963 birth cohort to 25.5% among 1994–1998 cohorts but a rise in FGC between 1994–1998 cohorts and 1999–2003 cohorts (28.4%). The percentage of respondents who circumcised their daughters reduced from 40.1% among the oldest birth cohort to 3.6% among the younger cohort. Birth-cohort, religion, education, residence, region, and ethnicity were associated with FGC. Factors associated with the daughter’s circumcision were birth-cohort, religion, residence, region, ethnicity, wealth, marital status, FGC status of the respondent, and FGC required by religion. Similar factors were found for discontinuation intention.

**Conclusions:**

The practice of FGC is still high but decreasing among younger birth-cohorts in Nigeria. There is no significant change in the perception of the discontinuation of FGC. More awareness about the adverse effects of FGC, particularly among women with poor education in Nigeria will greatly reduce this cultural menace’s timely eradication.

## Background

Female genital cutting (FGC) comprises all surgical procedures involving partial or total removal of the external genitalia and/ injury to the female genitalia organs whether for cultural or any other non-therapeutic reasons [1, 2]. An estimated 200 million girls and women have undergone FGC globally. The practice of FGC cuts across over 30 countries in Africa, Asia, and the Middle East [3]. In Africa, more than 101 million girls aged 10 years and above have undergone FGC [4], and an estimated 3 million girls have their genital cut annually [2, 5], as the practice is deeply entrenched in African culture [6]. The Practice of FGC is high in Nigeria with one-quarters of the global estimates occurring in the country, and it cuts across all socio-cultural and geo-political zones in the country [7]. The current national prevalence of FGC among women of reproductive age stood at 20 percent and 19 percent among daughters less than 14 years of age, with the highest prevalence among adult women (35%) found in the South East, followed by South West (30%) and lowest in the North East (6%) region of the country [8].

In Nigeria, the practice of FGC is performed during infancy especially within 8 days of delivery among some cultures, before marriage in some other societies most especially among the Ibo of the South of Nigeria, and sometimes before the birth of the first child in some other societies [3, 6, 9]. It is mostly performed by the traditional birth attendants and the local circumcisers with no medical training, using unsterilized instruments such as razor blades, scissors, and broken glasses [5, 10, 11], while some few health workers are also involved in the operation [3, 12, 13].

FGC is justified among the perpetuators on the premise that the procedure helps in preventing promiscuity, among women, initiates girls to womanhood, and promotes women’s chastity. It also increases male sexual pleasure, prevents infant and child deaths, enhances women’s fertility and child survival. Other reasons cited for the practice of FGC are cultural and religious beliefs even though the practice is independent of religion [7, 11, 14]. FGC has no health benefit but inflicts serious health complications which are irreversible on its victims; such as menstrual pain, excessive bleeding during delivery, infections (such HIV/Aids, hepatitis, urinary tract infections, abscesses, etc.), painful intercourse, and can also results into death of the victims through severe bleeding [11, 15]. It is also a major contributor to maternal and child deaths especially during delivery [16, 17]. The practice of FGC exerts social and psychological trauma on its victims [13, 14] and also constitutes the violation of fundamental human rights of both girls and women [18]. This is because it is commonly performed on infants when the individual consent is not given before it is performed [2, 19].

FGC is an unhealthy old cultural practice that must be unequivocally eradicated for the benefit of women and girls. The international agency and several governments have outrightly condemned the practice of FGC based on human rights violations and the associated health complications. The government of Nigeria has also joined some other parts of the world in making laws against the practice of FGC, for instance, the “Violence Against Persons Prohibition Act 2015” was passed in May 2015 [20]. The country was among the five countries calling for the eradication of FGC at the forty-sixth World Health Assembly [5]. Despite efforts aimed at eliminating the practice of FGC in Nigeria, the practice though has declined, is still unabatedly high beyond the expected target as emphasized by Sustainable Development Goals 3 and 5 [6]. There is, therefore, the need to investigate the contributory factors to the perpetuation of FGC in Nigeria so as to put an end to the practice.

Several studies have been done on the practice of FGC but some of these earlier studies [5, 20] were carried out on the prevalence of FGC while some examined intergenerational attitudes towards FGC [19, 21]. Other studies have combined both prevalence and intergenerational attitudes towards FGC [22, 23]. However, all of these studies focused on individual respondents rather than a cohort. Hence, we conceptualised this study with the view to providing a better understanding of socio-cultural mechanisms aimed at eradicating FGC in Nigeria. The specific objectives of this study are to carry out a cohort analysis of the state of the practice of FGC in the country; investigate the prevalence of FGC among daughters and mothers; and examine attitude towards its discontinuation of FGC between the years 2009 and 2018. The findings from this study will assist public health programmers and policymakers in designing prompt and appropriate interventional programmes targeting the eradication of FGC in Nigeria.

### Theoretical framework: the theory of cultural relativism

The theory of cultural relativism postulates that no culture is superior to another and, therefore, every culture must be respected, preserved, and be independent of external influence. Cultural relativism is exhibited in Africa culture most especially in the practice of harmful traditional practices such as the practice of FGC. In Africa, FGC is protected by culture; it is a practice that is passed down from older generations to the younger ones for a cultural reason. In some societies in Africa, FGC is practiced as a rite of passage of girls to womanhood [24–26], while in other societies in Africa, it is practiced to protect women’s chastity and protect them from being promiscuous [2, 27]. Seeing FGC as an aspect of African culture that must be sustained is one of the reasons why the practice continues in most African societies; therefore, efforts to abolish FGC must be handled with extra care so that it is not seen as a deliberate attempt to enforce foreign culture on African women. While African traditional culture should be preserved, it is pertinent to note that harmful traditional practice (such as FGC) that violates women’s and girls’ rights should be discouraged.

## Methods

### Study area

The study was carried out in Nigeria among women of childbearing age who had given birth to at least a daughter. Nigeria is regarded as the most populous country in Africa with approximately 201 million people [28], while women and girls constitute about 49.3% of the country’s population [29]. The country has more than 250 ethnic groups but is majorly stratified into the Hausa, the Igbo, and the Yoruba [30]; while the Hausas can be found in the North, the Igbos in the East, the Yoruba ethnic group occupy the western part of the country. The practice of FGC is widespread in the country with the exclusion of a few tribes [31, 32].

### Study design and data collection

The study used the Nigeria Demographic and Health Survey (NDHS) data collected in 2008, 2013, and 2018. NDHS is a nationally representative household sample survey designed to collect data on fertility, mortality, reproductive health, family planning, child health, nutrition, HIV/AIDS, malaria, FGC, and a host of other health-related topics [8]. The surveys specifically collect data on various aspects of FGC such as knowledge of FGC, the prevalence of FGC among mothers and daughters, types of FGC, age at FGC, persons who performed FGC, and attitudes towards FGC. The survey employed a stratified two-stage sampling procedure in collecting data from the households. We confirm that all methods were carried out by relevant ethical considerations, guidelines, and regulations (http://dhsprogram.com/data/available-datasets.cfm).

### Variable description

Three outcome variables were used. The variables were derived from the question of whether or not the: (1) respondent was circumcised, (2) the respondent circumcised her daughter, and (3) whether FGC should be discontinued or not. However, at the point of data analysis for each of the outcome variables, women with missing information on that particular outcome variable were excluded. Since the FGC practice is usually carried out within the first 5 years of life in Nigeria [3], the analysis based on the first question was restricted to four variables (place of residence, religion, ethnicity, and region) while that of the second and third questions were based on background characteristics of the respondents (place of residence, religion, ethnicity, and region, wealth index, education, partner’s/husband’s education, marital status, FGC required by religion, and FGC status of the respondent). The primary independent variable was “birth cohort”. The cohorts were created in groups of five, based on the year and month of birth. The century month code (CMC) approach was used [33]. Three rounds of Nigeria Demographic Health and Survey datasets were merged to obtain the cohorts. Consequently, nine 5-year birth cohorts were created, these include 1959–1963, 1964–1968, 1969–1973, 1974–1978, 1979–1983, 1984–1988, 1989–1993, 1994–1998, and 1999–2003.

### Data analysis

Data analyses were carried out at three different levels. The levels are univariate, bivariate, and multivariate levels. The frequency distribution of important variables was carried out at the univariate level, the chi-square distribution test of association was performed at the bivariate level, and logistic regression at the multivariate level. Variables that were not statistically significant at the bivariate level were not included in the final analysis.

The logistic regression was of the form:

$$\log \left( {{\text{p}}/\left( {1 - {\text{p}}} \right)} \right) =\upbeta _{0} +\upbeta _{1} {\text{x}}_{1} +\upbeta _{2} x_{2} + \cdots\upbeta _{m} {\text{x}}_{m} ;$$

where *p* indicates the probability of experiencing FGC and *βis* are the regression coefficients associated with the reference group and the *xi* are the explanatory variables.

The data were weighted to extrapolate the results to other areas not included in the survey as a result of complex sampling designs (cluster sampling) used during data collection. This was done to obtain nationally representative data for the provision of estimates of *FGC*’s prevalence and differentials.

## Results

### Distribution of respondents by FGC status

**Fig. 1:**
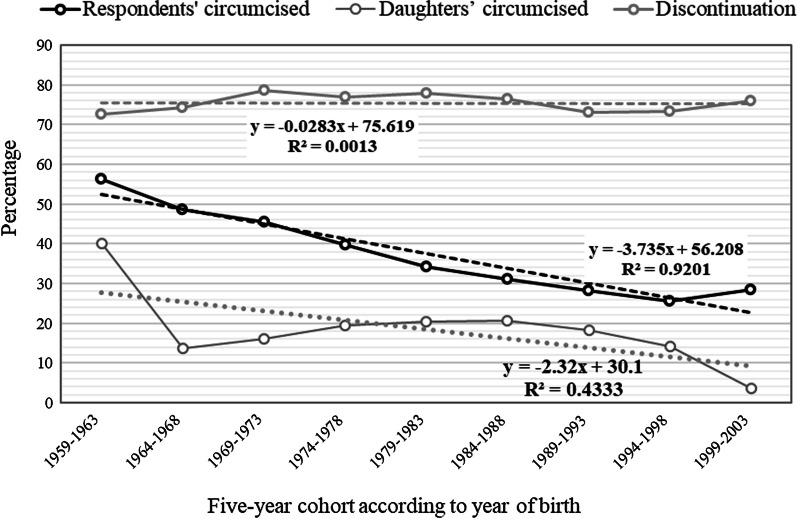
Five-year birth cohorts of circumcised women and daughters’ circumcision and discontinuation of the practice of FGC in Nigeria

The study found that that FGC has reduced over the years from 56.3% among the 1959–1963 birth cohort to 25.5% among 1994–1998 cohorts but a rise in FGC between 1994–1998 cohorts and 1999–2003 fellows (28.4%). The declining rate of FGC was − 3.735 (R^2^ = 0.9201). The percentage of respondents who circumcised their daughters reduced from 40.1% among the oldest birth cohort (1959–1964) to 3.6% among the younger cohort (1999–2003). Daughters’ circumcision increased steadily from 13.7% for the daughters of women in the second birth cohort (1964–1968) and peaked at 20.6% (1984–1988) and reduces consistently to 3.6% (1999–2003) (Fig. 1).

Table 1 showed that variables such as religion, ethnicity, and region were found to be significantly associated with FGC across the 5-year birth cohorts. The percentage of circumcised women was higher among Christian women in the 5-year cohort distributed from 1959 to 1993 but reduced among the 5-year cohorts that fall between 1994 and 2003. While 62.7% and 43.0% of women in the birth cohort 1959–1963 were circumcised in the Christian and Islam religious groups respectively, in the 1999–2003 birth cohort, a lower proportion of Christian (23.8%) women were circumcised compared to their Muslim (32.6%) counterparts. Igbo and Yoruba women demonstrated a higher rate of FGC than Hausa/Fulani ethnic groups.  Except for the earliest cohort (1959–1964), the percentage of circumcised women was higher among those who live in the urban area than those in the rural areas (Table 1).

**Table 1:** Distribution of circumcised women according to 5-year birth cohort by background characteristics

Background variables	5-year cohort according to year of birth								
	1959–1963	1964–1968	1969–1973	1974–1978	1979–1983	1984–1988	1989–1993	1994–1998	1999–2003
	n (%)	n (%)	n (%)	n (%)	n (%)	n (%)	n (%)	n (%)	n (%)
Total	966(56.3)	1297(48.7)	770(45.5)	683(39.7)	686(34.2)	676(31.2)	708(28.3)	596(25.5)	669(28.4)
Religion	73.2*	170.6*	85.6*	42.9*	13.7**	13.8**	***4.4***^***ns***^	***4.2***^***ns***^	25.2*
Christian	653(62.7)	873(59.7)	556(54.6)	431(46.7)	403(37.3)	377(34.9)	353(30.4)	246(24.5)	253(23.8)
Islam	263(43.0)	394(34.2)	210(31.7)	250(31.8)	282(31.0)	296(27.6)	351(26.6)	350(26.4)	415(32.6)
Others	50(78.1)	30(62.5)	4(36.4)	2(14.3)	1(6.7)	3(25.0)	4(23.5)	0(0.0)	1(7.1)
Ethnicity	304.5*	548.3*	351.6*	244.6*	233.9*	227.8*	238.7*	150.5*	130.9*
Hausa/F	103(35.6)	234(30.0)	117(27.3)	144(26.7)	165(27.2)	203(27.0)	247(26.3)	268(27.2)	317(33.7)
Igbo	317(80.5)	366(74.7)	318(67.7)	189(53.7)	196(48.8)	199(46.9)	205(44.0)	116(30.4)	142(32.0)
Yoruba	319(76.1)	422(80.1)	213(75.8)	221(71.3)	205(61.0)	178(55.1)	156(52.2)	143(45.7)	135(41.5)
Others	227(36.9)	275(31.8)	122(23.8)	129(24.8)	120(18.2)	96(14.5)	100(12.6)	69(10.5)	75 (11.7)
Region	400.6*	648.3*	388.7*	254.7*	54.9*	240.7*	239.2*	160.2*	136.0*
North Cent	88(41.9)	93(38.9)	53(27.5)	68(34.2)	70(24.6)	68(26.6)	72(20.9)	60(21.1)	55(21.1)
North East	9(4.5)	20(5.9)	21(8.1)	15(5.2)	28(7.4)	28(6.7)	52(9.3)	46(8.4)	53(11.9)
North West	104(42.3)	230(33.9)	98(30.6)	134(34.4)	141(35.1)	170(33.5)	190(31.9)	220(33.6)	268(39.1)
South East	287(82.9)	338(77.7)	292(68.9)	170(56.3)	179(53.8)	171(51.5)	183(49.1)	108(33.1)	124(33.1)
South South	183(58.7)	212(46.4)	106(46.3)	117(44.2)	85(30.8)	7 (23.7)	67 (22.5)	38 (17.5)	50(18.6)
South West	295(73.0)	404(78.6)	200(75.2)	179(63.9)	183(54.8)	164(48.8)	144(44.4)	124(39.9)	119(37.8)
Residence	***0.064***^***ns***^	78.3*	46.8*	54.9*	41.2*	34.0*	20.2*	5.0***	***0.016***^***ns***^
Urban	348(55.9)	693(58.3)	434(54.2)	381(49.4)	390(41.4)	383(37.4)	364(32.9)	271(27.9)	312(28.6)
Rural	618(56.5)	604(41.0)	336(37.7)	302(31.8)	296(27.8)	293(25.7)	344(24.7)	325(23.8)	357(28.3)

Bold values shows Chi square value of each variable

*n* number of cases of females circumcised within the group; *p < 0.001; **p < 0.01; ***p<0.05; *ns* not statistically significant at 5%

### Distribution of daughters by circumcision status

The distribution of circumcised daughters as reported by their mother is presented in Table 2. Across the birth cohorts, the data show that a higher proportion of Muslim women circumcised their daughters than their Christian counterparts. However, there is evidence that daughters’ circumcision has reduced over the years in each religious group. Among the Christians, the proportion of circumcised daughters reduced from 39.8% among 1959–1963 birth cohorts to 0.4% among 1999–2003 cohorts. This pattern was also observed among Muslim women. There was a consistent higher reporting of daughters’ circumcision among Hausa/Fulani women compared to Igbo and Yoruba women. A higher percentage of women in urban areas circumcised their daughters than those in rural areas. The daughters’ circumcision reduced from 39.4 to 40.5% among the birth cohorts 1959–1963 to 7.5% and 0.4% among the 1999–2003 cohorts. The proportion of circumcised daughters increased consistently with increasing levels of education. Daughters’ circumcision was found to be higher among women who claimed that FGC is required by religion and those who have undergone FGC.

**Table 2:** Distribution of circumcised daughters according to 5-year birth cohort by background characteristics

Background variables	5-year cohort according to year of birth								
	1959–1963	1964–1968	1969–1973	1974–1978	1979–1983	1984–1988	1989–1993	1994–1998	1999–2003
	n (%)	n (%)	n (%)	n (%)	n (%)	n (%)	n (%)	n (%)	n (%)
Total	607(40.1)	386(13.7)	283(16.1)	349(19.5)	431(20.5)	465(20.6)	477(18.2)	351(14.2)	91(3.6)
Religion	**4.41**	30.0*	47.1*	117.4*	169.5*	206.8*	217.1*	183.0*	65.4*
Christian	373(39.8)	158(10.6)	120(11.4)	98(10.2)	116(10.2)	97(8.6)	80(6.6)	35(3.3)	4(0.4)
Islam	204(39.3)	224(17.6)	163(23.4)	250(30.5)	315(33.0)	368(32.9)	397(28.7)	315(22.5)	87(6.4)
Others	30(53.6)	4(8.2)	0(0.0)	1(7.1)	0(0.0)	0(0.0)	0(0.0)	1(11.1)	0(0.0)
Ethnicity	192.9*	116.7*	104.0*	124.2*	185.5*	261.7*	273.9*	237.5*	72.4*
Hausa/F	79(32.8)	170(19.1)	130(28.9)	190(34.0)	242(38.1)	305(39.1)	335(34.3)	279(26.9)	75(7.5)
Igbo	189(53.5)	55(10.9)	71(14.5)	39(10.5)	62(14.2)	56(12.6)	46(9.5)	13(3.2)	2(0.4)
Yoruba	230(61.3)	120(22.1)	56(18.3)	67(19.9)	65(18.0)	57(16.0)	36(10.3)	19(5.2)	2(0.5)
Others	109(20.1)	41(4.7)	26(5.0)	53(10.0)	62(9.2)	47(6.9)	60(7.4)	40(6.0)	12(1.9)
Region	185.4*	119.1*	78.6*	126.9*	212.0*	243.2*	271.2*	240.5*	71.0*
North Cent	44(24.2)	20(8.3)	15(7.6)	30(14.8)	40(13.4)	36(13.6)	36(9.7)	17(5.6)	6(2.1)
North East	9(5.3)	11(3.2)	35(13.1)	62(20.9)	74(19.5)	97(22.9)	126(22.1)	100(18.0)	26(5.6)
North West	75(36.9)	168(21.4)	103(30.5)	151(37.7)	189(44.9)	222(42.2)	235(37.8)	202(29.3)	55(7.6)
South East	168(54.2)	52(11.6)	63(14.4)	36(11.4)	59(16.4)	53(15.2)	40(10.3)	12(3.5)	2(0.5)
South South	98(34.8)	29(6.3)	17(7.4)	21(7.8)	17(6.0)	8(2.5)	9(3.0)	4(1.8)	1(0.4)
South West	213(58.4)	106(20.1)	50(17.2)	49(15.9)	52(14.2)	49(13.2)	31(8.5)	16(4.5)	1(0.3)
Residence	0.172	192.9*	***0.094***	22.1*	20.2*	39.1*	75.8*	77.3*	42.9*
Urban	215(39.4)	161(13.0)	132(15.8)	117(14.6)	164(16.3)	162(15.0)	128(10.9)	74(7.0)	12(1.0)
Rural	392(40.5)	225(14.3)	151(16.3)	232(23.4)	267(24.3)	303(25.7)	349(24.1)	277(19.6)	79(5.9)
Education	17.4**	16.6*	38.2*	76.5*	156.1*	186.3*	208.7*	247.3*	102.4*
None	297(41.1)	201(15.7)	144(22.4)	203(29.5)	243(34.8)	280(35.6)	292(32.4)	224(28.1)	62(9.9)
Primary	192(44.0)	93(13.2)	63(12.7)	58(14.8)	71(19.0)	64(19.8)	46(15.1)	58(22.4)	12(5.0)
Secondary	86(39.1)	74(13.7)	64(15.1)	69(14.2)	101(14.8)	97(12.9)	125(12.5)	85(6.3)	17(1.1)
Higher	32(24.1)	18(6.5)	12(6.0)	19(8.3)	16(4.6)	24(6.1)	14(3.3)	4(1.1)	0(0.0)
Wealth Ind.	9.87***	26.8*	24.9*	79.3*	111.$*	127.5*	137.8*	190.3*	42.9*
Poorest	69(31.9)	94(18.3)	67(23.3)	112(34.6)	130(34.9)	160(37.5)	174(32.8)	146(29.6)	31(6.8)
Poorer	129(44.2)	79(15.1)	60(19.4)	71(22.3)	90(27.9)	107(28.7)	104(21.9)	97(20.3)	29(6.1)
Middle	146(41.5)	72(13.3)	63(15.1)	70(20.3)	88(22.9)	77(19.5)	91(18.1)	63(12.9)	20(3.8)
Richer	147(42.6)	96(14.3)	61(14.8)	49(13.5)	73(15.3)	84(17.5)	67(11.7)	31(5.6)	9(1.6)
Richest	116(37.8)	45(8.0)	32(9.6)	47(10.6)	50(9.1)	37(6.36.3)	41(7.6)	14(3.1)	2(0.4)
Marital Stat.	**5.94**	13.0**	7.725***	15.5*	33.3*	54.5*	108.8*	217.9*	278.4*
Never married	1(25.0)	0(0.0)	2(5.0)	0(0.0)	3(2.9)	4(2.2)	3(0.7)	3(0.3)	1(0.1)
Curr. married	491(38.9)	346(14.7)	238(17.2)	324(20.7)	411(22.3)	449(23.0)	462(21.9)	342(22.2)	89(14.6)
Separated/D	115(46.9)	40(9.3)	43(12.7)	25(13.2)	17(10.5)	12(9.5)	12(15.0)	6(10.0)	12(92.3)
Partner’s Ed.	24.2*	19.7**	20.2*	68.0*	103.0*	92.3*	93.9*	88.5*	**4.8**^**ns**^
None	235(40.1)	199(16.9)	22.9(103)	163(32.7)	180(36.9)	220(35.9)	238(34.1)	202(34.0)	54(17.1)
Primary	221(47.7)	88(13.1)	18.0(60)	49(17.4)	66(20.5)	53(20.3)	41(16.3)	29(15.8)	9(11.5)
Secondary	90(35.3)	56(11.5)	12.6(44)	79(16.1)	116(18.1)	123(18.2)	126(16.3)	71(12.9)	19(12.3)
Higher	56(29.6)	40(9.5)	12.0(28)	32(11.3)	41(11.0)	48(12.5)	51(13.9)	34(17.2)	5(9.3)
Don’t know	1(14.3)	2(28.6)	17.6(3)	1(10.0)	8(53.3)	5(31.2)	6(28.6)	6(50.0)	2(25.0)
CRR	143.0*	53.5*	107.5*	126.1*	169.9*	174.8*	220.0*	148.0*	82.7*
No	340(31.9)	234(11.7)	167(11.8)	211(14.8)	264(15.6)	272(15.3)	257(12.9)	187(9.9)	41(2.2)
Yes	192(71.9)	105(25.1)	103(36.7)	128(43.0)	158(46.6)	170(45.8)	207(41.1)	148(32.0)	48(11.0)
Don’t know	73(43.2)	47(12.6)	13(18.6)	10(13.3)	9(12.5)	23(20.9)	13(10.7)	16(13.6)	2(1.2)
CSR	649.7*	216.9*	118.6*	110.9*	232.5*	251.8*	215.4*	175.5*	34.4*
No	18(2.8)	54(4.0)	65(7.0)	116(11.2)	138(10.5)	166(11.2)	193(10.8)	148(8.5)	34(2.0)
Yes	589(67.9)	304(23.4)	204(26.5)	216(31.6)	270(39.4)	276(40.8)	253(35.7)	181(30.4)	46(6.9)

Bold values shows Chi square value of each variable

*n* number of cases of females circumcised within the group; *p < 0.001; **p < 0.01; ***p<0.05; *ns* Not statistically significant at 5%

### Attitude towards discontinuation of FGC

In Table 3, the data showed that Christian women consistently reported that FGC should be discontinued. The proportion of women that felt that FGC should be discontinued barely changes across the cohorts among Muslim and Christian women. In all the birth cohorts, a higher proportion of the Igbo and Yoruba women opined that the practice of FGC should be discontinued. However, the data showed that a higher percentage of the more recent cohorts of women wanted discontinuation of the FGC compared to the older cohorts, particularly among the Christian women. The proportion of women who had the notion that FGC should be discontinued was consistently higher among those who live in urban areas than their counterparts in rural areas. For instance, among the women in the birth cohorts 1999–2003, living in the urban and rural areas, 83.9% and 68.9% felt that FGC should be discontinued respectfully. Across all the birth cohorts, the proportion of women who said that FGC should be discontinued increases as the level of education increases. A similar pattern was observed by the husband/partner’s level of education. FGC discontinuation intention was more prevalent among uncircumcised women than those who were circumcised in each of the cohorts.

**Table 3:** Distribution of perception of women about FGC discontinuation according to 5-year birth cohort by background characteristics

Background variables	5-year cohort according to year of birth								
	1959–1963	1964–1968	1969–1973	1974–1978	1979–1983	1984–1988	1989–1993	1994–1998	1999–2003
	n (%)	n (%)	n (%)	n (%)	n (%)	n (%)	n (%)	n (%)	n (%)
Total	1073(72.7)	1864(74.4)	1271(78.5)	1268(77.0)	1516(77.9)	1567(76.4)	1761(73.2)	1637(73.3)	1713(75.9)
Religion	36.3*	101.3*	53.1*	167.3*	196.3*	234.3*	250.8*	236.9*	174.4*
Christian	714(77.8)	1148(82.1)	839(84.4)	809(88.9)	968(89.6)	971(89.9)	1022(88.0)	896(89.2)	954(88.3)
Islam	329(65.9)	691(65.1)	425(69.1)	447(61.8)	536(63.0)	587(61.1)	727(59.3)	735(60.2)	750(64.5)
Others	30(51.7)	25(54.3)	7(70.0)	12(85.7)	12(80.0)	9(81.8)	12(75.0)	6(66.7)	9(64.3)
Ethnicity	62.1*	99.8*	85.8*	185.9*	164.3*	264.1*	244.1*	245.7*	164.3*
Hausa/F	167(68.4)	485(65.7)	253(64.9)	278(55.9)	335(59.3)	368(54.8)	473(54.5)	501(55.7)	531(61.6)
Igbo	275(73.9)	376(78.2)	403(85.0)	321(89.7)	371(87.7)	376(87.4)	403(85.6)	346(88.7)	413(87.3)
Yoruba	212(59.6)	330(66.1)	191(71.0)	241(80.6)	267(81.7)	255(81.0)	245(82.5)	259(80.7)	250(79.1)
Others	419(83.3)	673(85.3)	424(87.2)	428(86.8)	543(86.1)	568(89.3)	640(83.2)	531(85.4)	519(85.8)
Region	47.3*	106.2*	76.5*	207.3*	186.6*	274.9*	262.4*	254.2*	157.9*
North Cent	141(77.5)	187(83.1)	160(86.5)	157(84.4)	234(85.7)	194(81.2)	256(77.1)	237(81.2)	215(83.7)
North East	135(90.0)	206(77.7)	189(80.1)	190(74.5)	252(75.0)	283(75.7)	373(71.9)	337(70.2)	290(72.5)
North West	141(66.2)	425(64.1)	188(63.1)	182(50.3)	207(53.6)	223(49.1)	276(48.9)	309(51.4)	376(59.8)
South East	246(74.1)	335(77.2)	368(85.4)	275(89.0)	303(86.8)	292(85.1)	326(85.3)	296(88.1)	351(87.1)
South South	196(76.0)	389(88.2)	185(86.0)	227(88.7)	241(88.9)	296(94.6)	262(88.8)	196(92.5)	232(89.2)
South West	214(62.9)	322(67.2)	181(71.3)	237(84.9)	279(84.3)	279(84.8)	268(85.6)	262(84.0)	249(81.1)
Residence	0.109^**NS**^	**3.5**^**NS**^	***2.3***^***NS***^	30.1*	43.9*	36.2*	78.9*	78.6*	69.8*
Urban	397(73.2)	862(76.1)	621(80.1)	621(83.2)	789(84.4)	820(82.2)	886(82.1)	794(82.9)	888(83.9)
Rural	676(72.5.)	1002(72.9)	650(77.0)	647(71.8)	727(71.9)	747(70.9)	875(66.0)	843(66.1)	825(68.9)
Education	24.1*	52.2*	66.8*	178.4*	222.2*	267.2*	256.7*	290.8*	192.9*
None	469(67.9)	735(68.1)	392(69.1)	360(59.3)	364(58.0)	367(54.6)	423(53.2)	346(50.6)	292(54.4)
Primary	313(74.0)	506(77.5)	400(85.3)	300(84.3)	290(84.1)	249(84.4)	220(77.5)	169(71.0)	164(74.2)
Secondary	164(76.6)	393(76.8)	303(76.9)	398(86.1)	556(86.5)	602(85.8)	772(82.0)	799(83.4)	1187(83.4)
Higher	127(86.4)	230(87.8)	176(93.1)	210(94.6)	306(92.7)	349(91.1)	346(89.9)	323(91.5)	70(93.3)
Wealth Ind.	**7.21**^**NS**^	84.3*	42.1*	155.1*	154.5*	177.7*	185.9*	244.0*	128.2*
Poorest	149(73.4)	239(59.2)	169(66.0)	153(52.6)	197(57.8)	212(55.6)	240(51.1)	211(48.6)	243(60.6)
Poorer	194(68.6)	320(69.7)	215(75.2)	203(71.7)	205(69.7)	213(65.9)	304(68.5)	280(64.8)	289(68.0)
Middle	243(70.8)	386(77.7)	323(83.0)	247(77.7)	271(76.3)	286(78.8)	359(76.7)	345(77.7)	362(76.1)
Richer	245(73.1)	488(77.0)	295(78.2)	292(87.2)	374(85.4)	362(83.2)	433(82.5)	427(84.6)	405(81.3)
Richest	242(77.8)	431(84.0)	269(86.5)	373(88.8)	469(90.5)	494(89.8)	425(85.3)	374(89.5)	414(90.8)
Marital Stat.	**0.120**^**NS**^	**5.61**^**NS**^	11.1**	6.64***	18.3**	27.5*	71.1*	153.2*	108.9*
Never mar.	10(76.9)	20(87.0)	35(89.7)	32(86.5)	83(90.2)	157(91.3)	364(90.1)	712(88.8)	1382(81.3)
Curr. Mar.	891(72.7)	1534(73.5)	975(76.8)	1087(76.0)	1301(76.4)	1313(74.5)	1341(69.7)	890(64.5)	323(59.4)
Separated/D	172(72.9)	310(78.1)	261(84.2)	149(83.2)	132(87.4)	97(82.9)	56(73.7)	35(67.3)	8(66.7)
Partner’s Ed.	30.1*	64.3*	33.5*	166.5*	240.1*	199.7*	174.9*	183.3*	29.0*
None	375(67.1)	660(66.5)	265(67.4)	242(54.8)	218(50.0)	276(52.5)	305(49.8)	216(42.4)	143(51.6)
Primary	328(72.2)	476(76.0)	257(80.8)	226(84.3)	253(83.2)	202(85.6)	182(76.8)	124(74.7)	38(54.3)
Secondary	186(75.6)	357(79.2)	255(78.2)	370(82.4)	505(84.6)	515(82.7)	571(79.6)	403(78.4)	100(70.4)
Higher	168(87.0)	339(85.4)	186(86.1)	242(92.0)	319(90.1)	314(85.8)	272(80.5)	144(80.0)	40(83.3)
Don’t know	3(75.0)	5(83.3)	12(70.6)	7(77.8)	6(50.0)	6(50.0)	11(52.4)	3(30.0)	2(28.6)
CRR	284.2*	503.1*	374.2*	310.0*	472.1*	486.1*	657.4*	627.4*	702.6*
No	899(84.0)	1571(84.1)	1144(87.3)	1124(85.7)	1379(87.0)	1408(86.3)	1571(85.1)	1467(85.4)	1540(88.0)
Yes	101(34.8)	118(30.0)	84(32.9)	102(36.8)	99(31.4)	106(30.8)	127(26.7)	114(26.1)	103(25.6)
Don’t know	71(62.8)	175(71.4)	43(79.6)	42(71.2)	38(82.6)	53(69.7)	63(74.1)	56(70.9)	70(68.0)
CSR	292.4*	132.1*	106.3*	76.7*	138.4*	198.7*	277.3*	302.4*	457.8*
No	595(96.0)	1021(84.9)	760(88.4)	812(84.7)	1060(86.0)	1166(85.5)	1385(82.9)	1340(63.1)	1384(88.3)
Yes	475(55.8)	779(64.6)	469(66.9)	414(65.8)	389(62.0)	345(56.5)	308(48.6)	230(44.4)	251(44.0)

Bold values shows Chi square value of each variable

*CRR* circumcision required by religion, *CSR* circumcision status of the respondent, *n* number of cases of females circumcised within the group; *p < 0.001; **p < 0.01, ***p<0.05; *ns* not statistically significant at 5%

### Relationship between FGC, daughter’s circumcision and opinion about its discontinuation and socio-demographic factors

Table 4 shows the unadjusted odds ratios of factors accounting for FGC among respondents, daughter’s circumcision, and opinion about its discontinuation of FGC in Nigeria. Statistical significant factors related to FGC were birth-cohort, religion, education, residence, region, and ethnicity. Factors associated with both the daughter’s circumcision and FGC discontinuation intention were birth-cohort, religion, residence, region, ethnicity, wealth, marital status, FGC status of the respondent, and if FGC was required by religion. Among 1959–1963 cohorts, the odds of FGC was 3.2 (2.83–3.69) higher compared to the youngest cohorts (1999–2003). A similar pattern was recorded for the daughter’s circumcision. However, there was no clear disparity in the pattern observed among the cohorts with respect to the notion of FGC discontinuation.

**Table 4:** Distribution of respondents by FGC status, daughter’s circumcision and opinion about its discontinuation and socio-demographic factors

Background	uOR (95% CI)	uOR (95% CI)	uOR (95% CI)
Characteristics	Respondent circumcised	Daughter’s circumcised	Circumcision discontinuation
Cohort			
1959–1963	3.2(2.83–3.69)*	7.7(4.08–12.5)*	1.1(0.93–1.26)
1964–1968	2.4(2.12–2.69)*	4.2(3.33–5.34)*	1.4(1.16–1.62)*
1969–1973	2.1(1.84–2.40)*	5.1(3.96–6.47)*	1.3(1.06–1.47)**
1974–1978	1.7(1.45–1.89)*	6.4(5.03–8.13)*	1.3(1.12–1.55)**
1979–1983	1.3(1.15–1.49)*	6.8(5.39–8.62)*	1.2(1.03–1.41)***
1984–1988	1.1(1.01–1.30)***	6.9(5.44–8.67)*	1.1(0.88–1.19)
1989–1993	0.9(0.87–1.13)	5.9(4.67–7.42)*	1.1(0.88–1.20)
1994–1998	0.9(0.75–0.98)***	4.4(3.46–5.58)*	1.2(1.01–1.37)***
1999–2003 (R.C)	1.0	1.0	1.0
Religion			
Christian	1.0	1.0	1.0
Islam	0.6(0.57–0.65)*	2.7(2.49–2.92)*	0.3(0.24–0.29)*
Others	1.2(0.90–1.58)	1.9(1.29–2.70)**	0.3(0.20–0.36)*
Ethnicity			
Hausa/F	1.0	1.0	1.0
Igbo	2.9(2.63–3.11)*	0.4(0.36–0.46)*	3.9(3.48–4.28)*
Yoruba	4.3(3.95–4.75)*	0.6(0.57–0.71)*	2.1(1.88–2.29)*
Others	0.6(0.58–0.69)*	0.2(0.19–0.24)*	4.2(3.79–4.56)*
Region			
North Central	1.0	1.0	1.0
North East	0.3(0.19–0.26)*	1.6(1.35–1.86)*	0.7(0.56–0.75)*
North West	1.4(1.24–1.56)*	3.6(3.14–4.22)*	0.3(0.24–0.31)*
South East	3.5(3.10–3.90)*	1.5(1.23–1.71)*	1.2(1.01–1.34)***
South South	1.4(1.27–1.62)*	0.7(0.59–0.88)**	1.6(1.39–1.93)*
South West	3.8(3.32–4.20)*	1.9(1.52–2.10)*	0.8()0.66–0.88*
Residence			
Urban	1.5(1.40–1.58)*	0.6(0.53–0.62)*	1.0
Rural	1.0	1.0	0.6(0.51–0.59)*
Education			
None		6.2(5.21–7.46)*	1.0
Primary		3.8(3.15–4.62)*	2.6(2.35–2.87)*
Secondary		1.9(1.60–2.34)*	3.2(2.97–3.51)*
Higher		1.0	6.8(5.89–7.97)*
Wealth Ind.			
Poorest		3.8(3.32–4.30)*	1.0
Poorer		2.8(2.43–3.17)*	1.7(1.50–1.85)*
Middle		2.1(1.87–2.45)*	2.6(2.30–2.85)*
Richer		1.6(1.43–1.88)*	3.3(2.96–3.66)*
Richest		1.0	5.2(4.65–5.87)*
Marital Stat.			
Never married		0.2(0.10–0.41)*	1.5(1.24–1.71)*
Curr. married		1.4(1.21–1.60)*	0.7(0.58–0. 76)*
Separated/D		1.0	1.0
Partner’s Ed.			
None		1.0	2.7(2.39–2.97)*
Primary		0.7(0.59–0.74)*	3.1(2.79–3.39)*
Secondary		0.5(0.43–0.53)*	4.6(4.07–5.28)*
Higher		0.4(0.32–0.42)*	0.9(0.64–1.45)
Don’t know		1.03(0.68–1.55)	1.0
CRR			
No		1.0	1.0
Yes		3.9(3.65–4.32)*	0.1(0.08–0.81)*
Don’t know		0.8(0.55–1.28)	0.5(0.35–0.72)*
CSR			
No		1.0	4.5(4.15–4.81)*
Yes		6.0(5.53–6.54)*	1.0

*CRR* circumcision required by religion, *CSR* circumcision status of the respondent, *n* number of cases of females circumcised within the group; *p < 0.001; **p < 0.01; ***p<0.05; *ns* not statistically significant at 5%

Table 5 presents adjusted odds ratios of factors associated with FGC among mothers, daughters, and opinions about the discontinuation of FGC. The identified predictors of FGC included: birth-cohort, religion, education, residence, region, and ethnicity. In 1959–1963, 1964–1968 and 1984–1988 birth-cohorts, the likelihood of FGC was 3.1 (CI 2.70–3.62, *p* < 0.001), 2.4 (CI 2.10–2.72, *p* < 0.001) and 1.2 (CI 1.02–1.35, *p* < 0.05) respectively, compared to 1999–2003 cohort. The earlier cohorts were more predisposed to the daughter’s circumcision than the later cohorts. For instance, the odds were 6.9 (CI 4.85–13.71, *p* < 0.001) times higher among 1959–1963 birth cohorts than those in 1999–2003 cohorts. Birth cohorts were not an identified predictor of FGC discontinuation.

**Table 5:** Adjusted odd ratios of factors associated with FGC among mothers, daughters and opinion about discontinuation of FGC

Background	aOR (95% CI)	aOR (95% CI)	aOR (95% CI)
Characteristics	Respondent circumcised	daughter’s circumcised	Circumcision discontinuation
Cohort			
1959–1963	3.1(2.70–3.62)*	6.9(4.85–13.71)*	1.2(0.92–1.66)
1964–1968	2.4(2.10–2.72)*	2.2(1.61–3.01)*	1.2(0.90–1.56)
1969–1973	2.1(1.78–2.38)*	3.1(2.23–4.29)*	1.3(0.97–1.75)
1974–1978	1.6(1.41–1.89)*	4.3(3.12–5.88)*	1.3(0.96–1.71)
1979–1983	1.3(1.16–1.54)*	5.7(4.17–7.79)*	1.1(0.81–1.43)
1984–1988	1.2(1.02–1.35)***	5.8(4.24–7.88)*	0.9(0.75–1.31)
1989–1993	1.1(0.96–1.26)	4.6(3.36–6.20)*	0.8(0.63–1.09)
1994–1998	0.9(0.79–1.05)	3.7(2.68–5.01)*	0.7(0.58–1.02)
1999–2003 (R.C)	1.0	1.0	1.0
Religion			
Christian	1.0	1.0	1.0
Islam	1.5(1.31–1.67)*	1.5(1.27–1.81)*	0.6(0.51–0.73)*
Others	1.3(0.97–1.86)*	0.6(0.39–1.03)	0.8(0.48–1.16)
Ethnicity			
Hausa/F	1.0	1.0	1.0
Igbo	0.8(0.60–0.97)*	0.9(0.58–1.29)	1.3(0.84–1.95)
Yoruba	2.4(1.97–2.99)*	1.3(0.97–1.74)	0.8(0.57–1.05)
Others	0.4(0.33–0.47)*	0.5(0.42–0.61)*	1.2(0.97–1.41)
Region			
North Central	1.0	1.0	1.0
North East	0.2(0.16–0.24)*	3.1(2.36–3.95)*	0.6(0.50–0.81)*
North West	0.9(0.75–1.06)	3.4(2.64–4.40)*	0.5(0.35–0.58)*
South East	3.9(3.24–4.89)*	1.3(0.86–1.92)	1.2(0.80–1.85)
South South	2.5(2.20–2.94)*	1.1(0.78–1.37)	1.4(1.10–1.89)**
South West	1.5(1.31–1.79)*	1.2(0.87–1.43)	0.9(0.71–1.18)
Residence			
Urban	1.1(1.05–1.22)*	0.8(0.70–0.89)*	1.0
Rural	1.0	1.0	0.9(0.87–1.12)
Education			
None		1.8(1.37–2.37)*	1.0
Primary		1.9(1.42–2.40)*	1.2(1.01–1.38)***
Secondary		1.7(1.32–2.12)*	1.2(0.97–1.39)
Higher		1.0	1.9(1.40–2.48)*
Wealth Ind.			
Poorest		1.8(1.46–2.26)*	1.0
Poorer		1.4(1.12–1.71)**	1.4(1.18–1.59)*
Middle		1.4(1.14–1.68)**	1.7(1.46–2.05)*
Richer		1.2(1.04–1.48)***	1.9(1.52–2.24)*
Richest		1.0	2.1(1.63–2.58)*
Marital Stat.			
Never married		0.7(0.61–1.15	1.3(1.14–1.69)*
Curr. married		0.9(0.71–1.16)	0.5(0.38–0. 57)*
Separated/D		1.0	1.0
Partner’s Ed.			
None		1.0	
Primary		1.1(0.93–1.29)	1.4(1.15–1.59)*
Secondary		1.1(0.93–1.27)	1.3(1.07–1.49)**
Higher		1.2(0.92–1.38)	1.5(1.22–1.86)*
Don’t know		1.6(0.97–2.78)	0.6(0.31–0.98)***
CRR			
No		1.0	
Yes		3.2(2.88–3.59)*	0.1(0.07–0.10)*
Don’t know		0.8(0.45–1.29)	0.8(0.48–1.19)
CSR			
No		1.0	7.9(7.03–9.07)*
Yes		6.1(2.94–9.39)*	1.0

*CRR* circumcision required by religion, *CSR* circumcision status of the respondent, *n* number of cases of females circumcised within the group; *p < 0.001; **p < 0.01; *ns* not statistically significant at 5%

## Discussion

Female genital cutting is a harmful traditional practice that is yet to be fully addressed in developing countries despite public health interventions and programmes instituted by governments and international agencies toward its eradication [8, 33]. The practice of FGC is high and widespread in Nigeria and cuts across various socio-economic groups in the country. To assess the compliance with the interventional programmes aimed at eradicating this unacceptable practice in Nigeria, it is thus essential to examine the intergenerational attitudinal change to FGC. In this study, 5-year birth cohorts of women from 1959 to 2003 were used to address this important public health challenge in Nigeria.

In this study, we found that the practice of FGC has declined consistently over the years. There is evidence that the younger cohort of women exhibited a reduction in the level of FCG practice compared to older cohorts This finding was in agreement with the outcomes of similar studies conducted in some parts of sub-Saharan Africa and other parts of the world [21, 34–36]. Our findings point to the importance of public health campaigns against FGC in Nigeria. Although passive, the enactment and implementation of national and state policies [19] on FGC in Nigeria could also be responsible for the change.

The level of daughters’ circumcision in society is evidence of intergenerational change to this harmful traditional practice. It also shows the effectiveness of the strategies in place aimed at eradicating this social menace [1, 3, 5]. In this study, although the level of the daughter’s circumcision shows a declining trend, we also found that the practice of FGC among daughters remained more prevalent among circumcised mothers compared to their uncircumcised counterparts. These outcomes corroborate the findings from earlier studies [6, 34, 37] This might be unconnected with the transfer of cultural legacy to the younger generations.

We also found no clear distinction about the intention to discontinue FGC among the birth cohorts but found that the FGC status of women has a great influence on the opinion to discontinue the practice of FGC. More circumcised women believed that the practice should be discontinued. This might be ascribed to the fact that the circumcised women have realized the health implications of FGC due to an on-going campaign against the practice. However, this finding was in contrast to the study by Gbadebo [19] that established that uncircumcised women wanted the practice of FGC discontinued.

This study found religion, ethnicity, region, and place of residence to be among the principal factors accounting for cohort disparity in the practice of FGC in Nigeria. In spite that the practice of FGC has no religious connection, this study unveiled that more Christians have undergone FGC and were more likely to circumcise their daughters than their Moslems counterparts. This has also been documented in the studies conducted by [7] and Alo and Gbadebo [23]. Different ethnic groups have diverse peculiarities towards some traditional practices. In this study, the prevalence of FGC was higher among the Yoruba women than their counterparts from other ethnic groups. Generally, Yorubas were more educationally advantaged than the other ethnic groups; their attitudinal change towards the discontinuation of FCG might be due to their high level of educational attainments. Other earlier studies [9, 22, 23] have also reported similar outcomes. The practice of FGC was also found to be more prevalent in the South-West and South-East regions of the country than in any other regions. This was similar to the findings by [38, 39] Several other studies [5, 20, 22] have revealed that FGC is more prevalent in rural areas than urban; this was also corroborated by this study, where a large number of women in rural areas have been circumcised, circumcised daughters and want the practice continued than their counterparts in urban areas. However, this was contrary to the findings of Epundu [9] which reported a higher prevalence of FGC in urban areas, which might have arisen due to rural–urban shift.

## Study limitations

The data used is retrospective with the possibility of recall bias. The study design was cross-sectional, therefore only association could be established. Cautions must therefore be exercised by readers because the findings of the association between the outcome variables and the explanatory variables did not infer causality. Also, there was some missing information. Nonetheless, the use of nationally representative data collected over 3 waves and the rigorous procedure adapted by the data owners in screening information collected from respondents.

## Conclusion

The practice of FGC is still high but decreasing among younger birth-cohorts in Nigeria. We found variability in the prevalence and factors associated with respondents’ circumcision, daughters’ circumcision, and attitude towards the discontinuation of FGC across the different cohorts included in this study. The practice of FGC cuts across religious groups, level of education, ethnic diversities, and the FGC status of the woman. This harmful traditional practice remains a problem in the country as it transcends from one generation to the other. There is no significant change in the perception of the discontinuation of FGC. More awareness about the adverse effects of FGC, particularly among women with poor education in Nigeria, will significantly contribute to the timely eradication of this cultural menace. More proactive measures about the eradication of FGC are needed for the timely eradication of this cultural menace. However, future research should consider a prospective cohort study of FGC among younger women of reproductive age groups; this could provide additional information towards the eradication of FGC in Nigeria.

## Data Availability

The data used for this article is available at http://dhsprogram.com/data/available-datasets.cfm. For further information on the use of the data, contact: The DHS Program Office, ICF, 530 Gaither Road, Suite 500, Rockville, MD 20850 USA, Tel: + 1 (301) 407-6500, Fax: (301) 407-6501.
